# Post‐stroke cognitive deficits rarely come alone: Handling co‐morbidity in lesion‐behaviour mapping

**DOI:** 10.1002/hbm.24885

**Published:** 2019-11-29

**Authors:** Christoph Sperber, Chloé Nolingberg, Hans‐Otto Karnath

**Affiliations:** ^1^ Centre of Neurology, Division of Neuropsychology, Hertie‐Institute for Clinical Brain Research University of Tübingen Tübingen Germany

**Keywords:** lesion‐deficit inference, nuisance regression, principal component analysis, statistical parametric mapping, VLSM

## Abstract

Post‐stroke behavioural symptoms often correlate and systematically co‐occur with each other, either because they share cognitive processes, or because their neural correlates are often damaged together. Thus, neuropsychological symptoms often share variance. Many previous lesion‐behaviour mapping studies aimed to methodologically consider this shared variance between neuropsychological variables. A first group of studies controlled the behavioural target variable for the variance explained by one or multiple other variables to obtain a more precise mapping of the target variable. A second group of studies focused on the shared variance of multiple variables itself with the aim to map neural correlates of cognitive processes that are shared between the original variables. In the present study, we tested the validity of these methods by using real lesion data and both real and simulated data sets. We show that the variance that is shared between post‐stroke behavioural variables is ambiguous, and that mapping procedures that consider this variance are prone to biases and artefacts. We discuss under which conditions such procedures could still be used and what alternative approaches exist.

## 1 INTRODUCTION

Mapping stroke patients' brain lesions is a powerful method to investigate functional brain anatomy, and voxel‐based lesion‐behaviour mapping (VLBM) has become an outstandingly popular method in this field (Bates et al., 2003; Karnath et al., 2018; Rorden & Karnath, 2004). In recent years, over a hundred studies utilised this statistical method to map the anatomy of cognitive functions (Karnath & Rennig, 2017). Many of these studies investigated cognitive disorders that consist not only of a single disturbed symptom, but are linked to further cognitive or neurological (sub‐)deficits. For example, several (partly) dissociating deficits of higher order motor skills are attributed to the term “apraxia” (e.g., Goldenberg & Randerath, 2015; Manuel et al., 2013; Martin et al., 2016), and patients with apraxia often also suffer from additional aphasia (Kertesz & Hooper, 1982; Papagno et al., 1993). Likewise, besides a neurological core symptom of ego‐centred spatial inattention in spatial neglect (Karnath 2015), other spatial and non‐spatial neuropsychological deficits may exist (Chechlacz et al., 2010; Husain & Rorden, 2003; Verdon et al., 2010; Vossel et al., 2011). Patients with a neuropsychological disorder may also suffer from additional primary motor deficits, such as pusher syndrome (Karnath, 2007), or from additional primary visual defects, as in pure alexia (Pflugshaupt et al., 2009). The neural correlates of such co‐occurring and correlating symptoms and how they relate to each other have been the subject of many lesion‐behaviour mapping studies. Importantly, the multifactorial character of such post‐stroke deficits is not limited to cognitively related symptoms. Due to proximity of distinct neural correlates and the typical vascular topography of stroke lesions, symptoms that do not share any common cognitive function nevertheless can correlate and share variance. Several lesion‐behaviour mapping studies explicitly aimed to capture aspects of this shared variance between behavioural symptoms. In the present manuscript, we focus on two strategies that are commonly used in VLBM studies: control for the variance of co‐occurring symptoms and mapping only the shared variance of behavioural variables.

A plethora of VLBM studies utilised nuisance regression or similar approaches to control the variable of interest for the variance explained by additional behavioural variables (e.g., Almairac et al., 2015; Baldo et al., 2013, 2018; Finkel et al., 2018; Gajardo‐Vidal et al., 2018; Jones et al., 2016; Lorca‐Puls et al., 2018; Martin et al., 2017; Moon et al., 2018; Pillay et al., 2014, 2017; Schwartz et al., 2011; Walker et al., 2011; Wilson et al., 2015; Winder et al., 2016). While some previous studies provide a rationale for covariate control, others do not provide any rationale at all or simply resort to a data driven procedure, where all known variables that correlate with the target symptom are controlled for. Generally, we see different rationales to use such a strategy of covariate control. First, one might expect that not only the behaviour of interest affects the measured variable, but that other confounding factors directly influence the measured variable as well. A second reason could be that researchers observe a correlation between behavioural scores in their experiment, while actually no shared neural correlates are expected. In this situation, shared variance likely occurs due to proximity of neural correlates and systematic collateral damage due to stroke. A VLBM therefore might not only map the cognitive process of interest, but—to a smaller extent—also the confounding cognitive or neurological function. Thus, variance explained by the confounding variable is removed, and the resulting variable is seen as a putatively “pure” measure of the behavioural variable under interest. A third rationale is that a researcher expects partially shared neural correlates of two variables, and that the VLBM analysis is intended to only identify brain areas that exclusively underlie one of these behavioural variables. In this situation, controlling for the second variable is thought to ensure that topographical results are only related to brain areas that are exclusive to the main target variable.

Another branch of VLBM studies did not remove the shared variance of behavioural symptoms, but instead focused on this shared variance. The idea is that multiple behavioural variables exist that share cognitive processes, and that the shared variance of these behavioural variables represents these cognitive processes. In the example of aphasia, one could aim to identify common factors in a test battery that represent certain fundamental cognitive processes such as semantic or phonological abilities. Shared factors of correlating variables can be found by dimension reduction techniques, for example, factorial analysis or principal component analysis, and several VLBM studies mapped such common factors (e.g., Aguilar et al., 2018; Butler et al., 2014; Chechlacz et al., 2014; Chen et al., 2016; Fridriksson et al., 2016; Halai et al., 2017; Lau et al., 2015; Timpert et al., 2015; Tochadse et al., 2018; Verdon et al., 2010).

**Figure 1:**
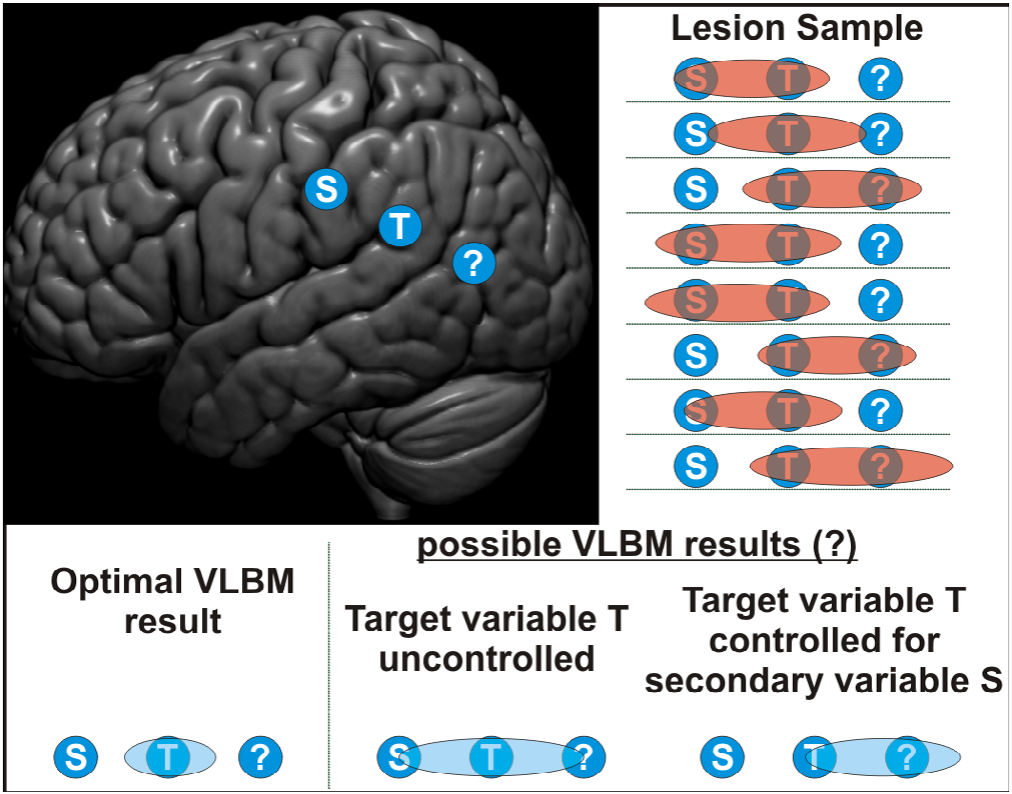
Illustration of a possible bias in voxel‐based lesion‐behaviour mapping (VLBM) controlled for a secondary variable. Damage to area T induces the behavioural target behaviour that we want to map in the analysis. A secondary behavioural variable correlates with the target variable; damage to the secondary behaviour's neural correlate S induces this deficit. Both areas T and S are often damaged together. Therefore, an uncontrolled VLBM analysis might not only find area T, but also area S. An imaginable bias when we now control the target variable for the secondary variable is that the VLBM might find areas that are usually damaged together with area T, but not with S. This is the ‘?’ area. The example is chosen arbitrarily and is not intended to correspond to any real study

However, there are potential pitfalls in the methodological strategies outlined above. First, some uncertainties about the relation of two behavioural variables might exist—correlation between both might originate from proximity of neural correlates, or from different degrees of overlap. For many behavioural deficits, the scientific community offers no consensus on this question. Second, and more relevant, variables obtained after control by nuisance regression or shared factors might not necessarily represent what they are supposed to. What covariates to include into statistical models is a discussion with a long history. Recently, in several scientific fields such as organisational psychology, complex problems with variable control have been discussed critically (Becker, 2005; Breaugh, 2006, 2008; Newcombe, 2003). These problems include, among others, that the control of covariates is often not explained by researchers, that control strategies are often employed in a too naïve way, or, in the worst case, that control of covariates might even remove the actual effect instead of biases. A deeper theoretical background to covariate control can be provided by the causal inference literature (Pearl, 1996; Pearl & Mackenzie, 2018). Using causal models, situations can be identified either where covariate control should be implemented to obtain valid inference, or where covariate control is in fact detrimental to the investigation of causal relations between variables. Problems with the control of covariates are likely more eminent in lesion mapping, where data are comprised of many layers of complexity. For example, if shared variance between two symptoms exists, we might find a common factor that is not related to shared neural correlates of both behaviours, but instead to brain areas that might be damaged together with the neural correlates of both functions regularly. Furthermore, it is imaginable that controlling for a second variable might misplace significant findings away from the origin of the signal (Figure 1), if control for covariates unintentionally removes parts of the actual effect that is investigated. Such bias might further be mediated by lesion size, as larger lesions often induce stronger deficits, but also do more collateral damage.

The present study aimed to investigate the validity of the above described strategies to capture aspects of shared variance of behavioural variables in lesion‐behaviour mapping. We performed VLBM using such strategies in lesion samples either mapping well‐controlled simulated or real behavioural data. We hypothesised that, given both the complexity of multivariate post‐stroke behaviour and the complexity of brain damage patterns, such strategies might lead to artefacts, that is, biased results or false positive findings.

## 2 METHODS

Brain imaging data of acute, first‐ever right hemisphere stroke patients admitted to the Centre of Neurology at the Tübingen University Hospital were included. The study has been performed in accordance with the standards laid down in the revised Declaration of Helsinki and patients or their relatives consented to the scientific use of the data.

Structural lesion images were acquired either by CT or MRI. If both modalities were available, MRI was preferred. For MRI, we used either diffusion‐weighted imaging in the first 48 hr after stroke, or T2 fluid attenuated inversion recovery images at later stages. Binary lesion maps were manually delineated on transversal slices of the image using MRIcron (https://people.cas.sc.edu/rorden/mricron/index.html). Lesion maps were then normalised into 1 × 1 × 1 mm^3^ MNI space using SPM (https://www.fil.ion.ucl.ac.uk/spm) and the Clinical Toolbox (Rorden et al., 2012).

Lesion‐behaviour mapping was performed with NiiStat software (https://www.nitrc.org/projects/niistat/). In case that a VLBM was controlled for the effect of lesion size, this was done by regressing out lesion size from the behavioural variable in a nuisance regression (Karnath et al., 2004). All statistical analyses were performed using SPSS 25, further analyses and simulations were performed using custom scripts in MATLAB 2018 and SPM12.

## 3 EXPERIMENT 1: CONTROLLING FOR CO‐OCCURRING BEHAVIOURAL VARIABLES

### 3.1 Methods

**Figure 2:**
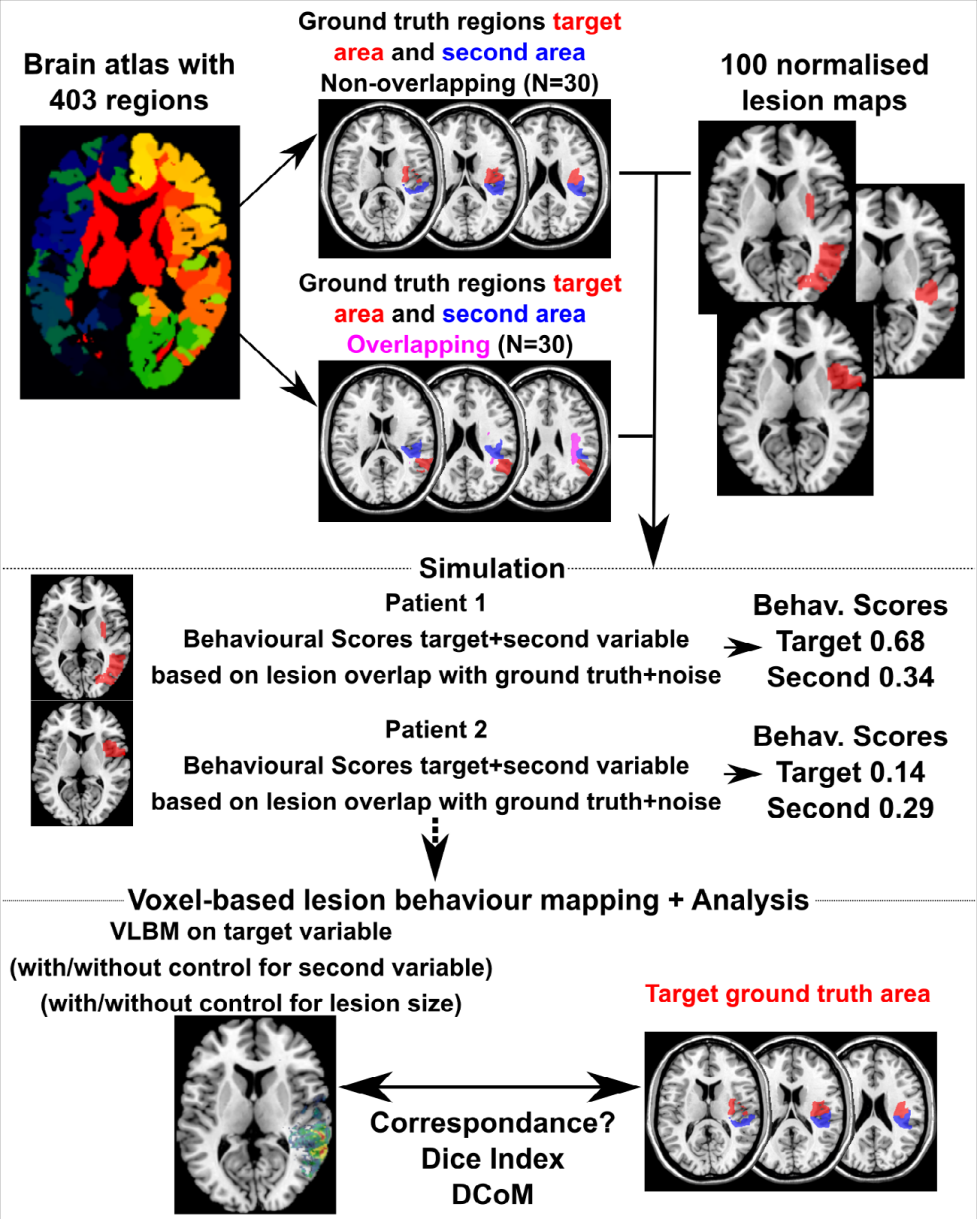
Experimental pipeline in Experiment 1

The first experiment aimed to evaluate different possible strategies in VLBM in the case of systematically co‐occurring symptoms. Two options were tested: control of the target variable by a second variable, and control by lesion size. This was carried out by employing a simulation approach in which, instead of real behavioural symptoms, simulated behavioural symptoms were used. These symptoms were simulated using real lesion images and arbitrarily chosen ground truth regions. These ground truth regions can be thought of as the neural correlates of the behaviour, which we have perfect knowledge of in the simulation setting. The quality of a VLBM method can then be tested by mapping the neural correlates of the simulated behaviour and comparing the results with the ground truth region (see Sperber & Karnath, 2018, for further details). A valid VLBM method should lead to results that highly correspond to the ground truth region; unsystematic or even systematic deviations suggest that the VLBM has limitations. The simulation approach in the present study is illustrated in Figure 2.

In detail, imaging data of 100 patients that were part of previous studies were used (Sperber & Karnath, 2017; Sperber et al., 2019). This sample size was chosen because it is in the typical range of real VLBM studies, but at the upper end where results are more stable than in small samples (see Lorca‐Puls et al., 2018). Ground truth regions were selected from a brain atlas that consisted of 403 brain regions (Pustina et al., 2018), which included 360 diluted regions taken from a multimodal grey matter parcellation (Glasser et al., 2016), as well as several subcortical and white matter areas. For each simulated symptom, we arbitrarily chose ground truth regions consisting of one, two, or three adjoined regions in this atlas. The aim of the simulation was to investigate how lesion‐behaviour mapping should be performed when two symptoms often co‐occur. Therefore, for each simulation run, two ground truth regions that had potential to lead to co‐occurring symptoms were chosen, that is, regions which might often be damaged together were chosen; boundaries of cerebral main artery territories were taken into consideration. One simulated behaviour was chosen to underlie the target behaviour and the other to underlie the second behaviour. Two different conditions were investigated: In the non‐overlap condition, the ground truth regions were lying in close proximity, but they were completely distinct without any overlap. In the overlap condition, the ground truth regions were partially overlapping in a way that each ground truth consisted partially of some exclusive region and partially of a region that was a shared neural correlate of both ground truths. For each condition, 30 target ground truth areas together with corresponding second ground truth areas were chosen.

Behavioural variables were simulated for each patient and for each ground truth based on a part of true signal and a part of noise to obtain more realistic behavioural variables. The procedure followed previous work that investigated how realistic signal in VLBM analyses compares to the signal in simulations given different simulation parameters (Pustina et al., 2018). Scores ranged from 0 (no symptom) to 1 (maximum symptom severity). The amount of noise was varied to obtain behavioural scores with correlations of different strength. For a first analysis, a maximum of 0.5 points was calculated as a linear function of damage of the lesion to the ground truth region, for example, 0 when a lesion did not affect the ground truth region and, for example, 0.5 × 0.25 = 0.175 when a lesion affected 25% of the ground truth region. A maximum of 0.5 points was drawn randomly, uniformly between 0 and 0.5. In the second analysis, maximally 0.7 points were given for true signal, and 0.3 points for noise, in order to obtain a higher correlation between symptoms. Sanity checks of simulated symptoms showed that symptoms were indeed systematically co‐occurring with a medium correlation in the first condition with 50% signal (average *r* = .38, SD = 0.12) and a high correlation in the second condition with 70% signal (average *r* = .69, SD = 0.12). Over all conditions, symptoms and lesion size correlated on average with *r* = .45.

VLBM analyses were performed using *t* tests with family‐wise error correction by maximum statistic permutation thresholding with 1,000 permutations at *p* < .05. Only voxels damaged in at least 10 patients were included in the analysis. Two experimental factors were varied in the VLBM analyses: (a) control for the second behavioural variable by nuisance regression, and (b) control for the effect of lesion size. Both variables were either controlled for or not controlled for, resulting in a 2 × 2 design.

Two dependent variables based on the binary VLBM maps and the maps of ground truth regions were tested. First, the Dice‐Index was computed. This is a measure of spatial congruency for binary maps. It ranges from 0 when no overlap between both maps exists to 1 if both maps perfectly overlap. Second, the distance between centres of mass (DCoM) of both maps was computed as in previous studies (Mah et al., 2014; Sperber & Karnath, 2017). This variable indicates how much the statistical map is spatially misplaced from areas where the true signal originated. A good VLBM method should yield a high Dice index and low DCoM. Statistical analyses were independently conducted for three situations: (a) non‐overlapping ground truth regions, (b) overlapping ground truth regions with a focus on the entire target region, and (c) overlapping ground truth regions with a focus only on the area that is exclusively underlying the target behaviour, but not the control behaviour. Although both factors were repeated measure factors, a standard repeated measures analysis was not feasible, because some analyses yielded non‐results. This resulted in empty cells and thus only partially paired data. With a multifactorial design and mixed model analyses being inadequate in this situation, we analysed the data with independent measure 2 × 2 ANOVAs, mirroring the analysis in a previous study (Sperber & Karnath, 2017).

### 3.2 Results

**Figure 3:**
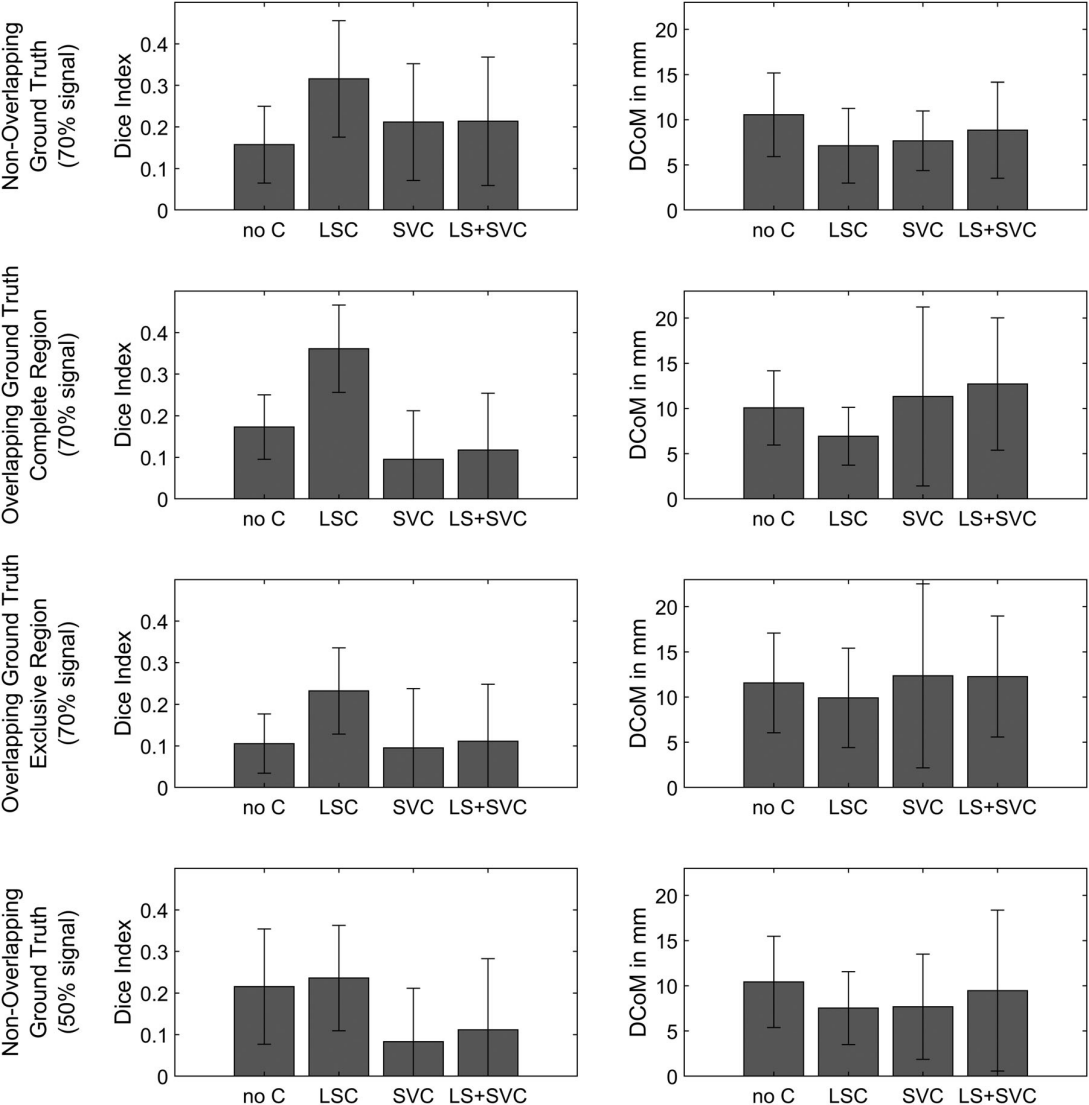
Results of Experiment 1, showing results for the four conditions: no control (no C), lesion size control (LSC), second variable control (SVC), and lesion size and second variable control (LS+SVC). Error bars indicate standard deviation. DCoM, distance centres of mass

**Figure 4:**
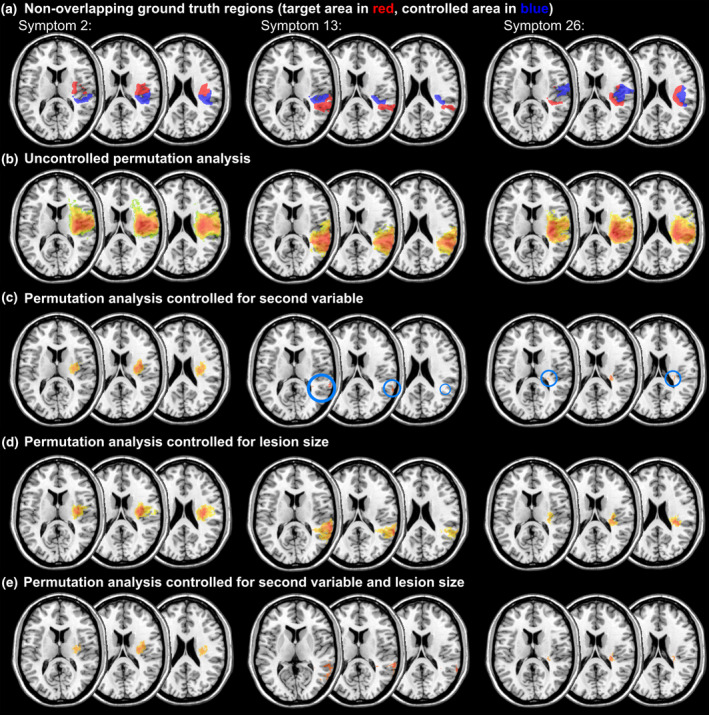
Example results of Experiment 1 for non‐overlapping ground truth regions in the condition with 70% signal. Statistical maps show statistically significant voxels after maximum statistic permutation control. Colour grading indicates *t*‐values, with warm red colours indicating higher values. Light blue circles have been added to highlight smaller clusters of significant voxels. a) Ground truth regions for the target symptom (red) and the secondary symptom (blue); b)‐e) permutation‐thresholded VLBM results for b) uncontrolled analysis; c) analysis controlled for the secondary variable; d) analysis controlled for lesion size; e) analysis controlled both for the secondary variable and lesion size

**Figure 5:**
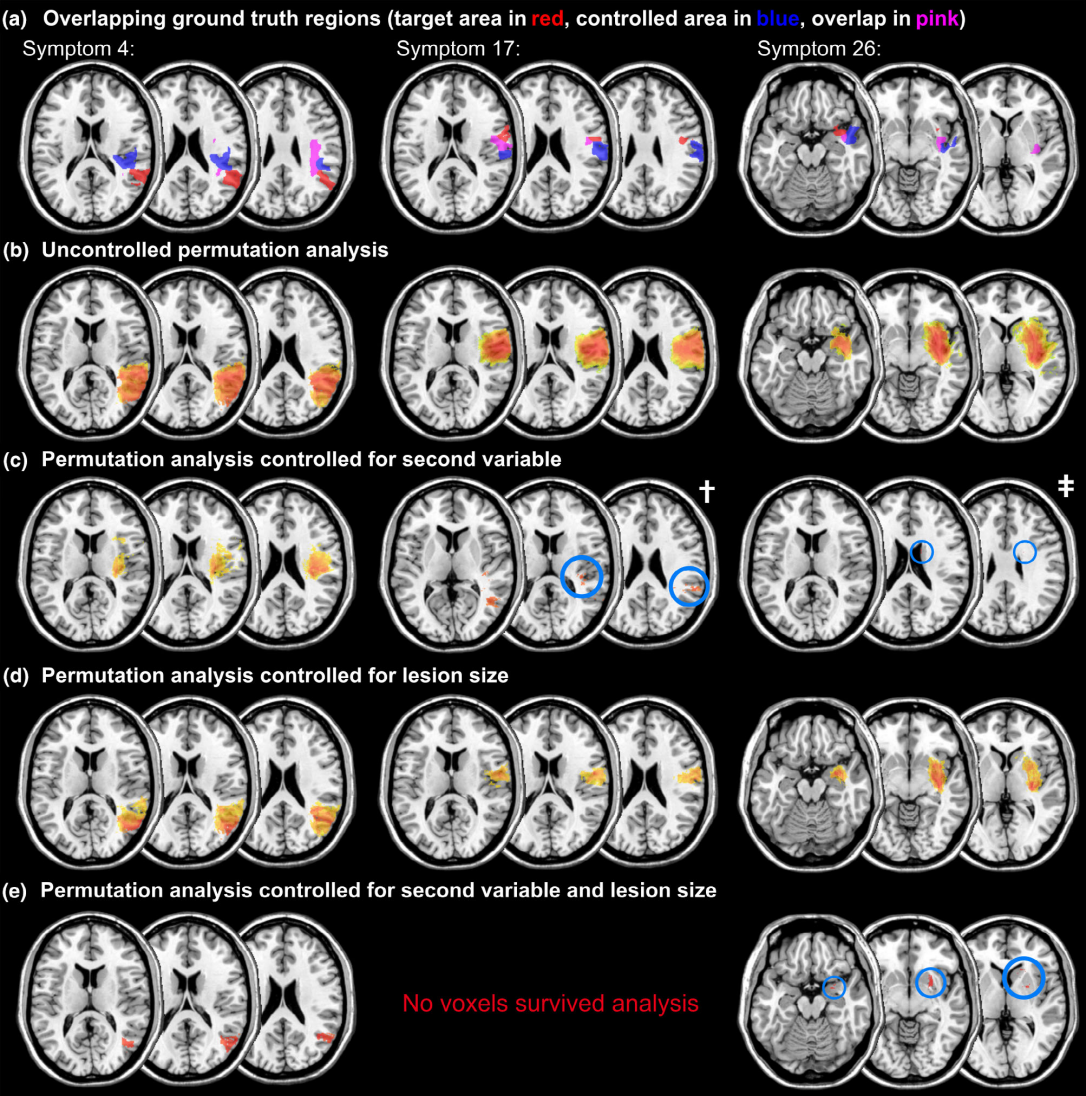
Example results of Experiment 1 for overlapping ground truth regions as in Figure 4. †The analysis in (c), middle column, did not yield positive, but only negative significant findings. These are shown here, but have been removed from the analysis in Experiment 1. ‡The analysis in (c), right column, did not yield significant voxels in the slices shown in the other panels, therefore the shown slices were changed to more anterior slices with positive findings. a) Ground truth regions for the target symptom (red) and the secondary symptom (blue), as well as overlap of both (pink); b)‐e) permutation‐thresholded VLBM results for b) uncontrolled analysis; c) analysis controlled for the secondary variable; d) analysis controlled for lesion size; e) analysis controlled both for the secondary variable and lesion size

In both conditions, several analyses did not yield any significant positive topographical results (Table 1). Non‐findings were almost exclusive to analyses that involved the control of the second variable via nuisance regression. In the condition with 50% signal and overlapping ground truth regions, nearly all analyses yielded non‐results. This condition was thus removed from further analysis. Moreover, in a few of these analyses there were not only no positive findings, but negative findings, that is, voxels where damage was significantly related to less severe symptoms. Visual sanity checks of these analyses revealed that these still found (non‐significant) positive signal around the ground truth area, but areas further away demonstrated significant negative findings. Note that these cases were counted as non‐findings and thus removed from further analysis. Results of 2 × 2 ANOVAs are visualised in Figure 3. Example results are shown in Figure 4 for non‐overlapping ground truths, and in Figure 5 for overlapping ground truth regions.

**Table 1:** Number of analyses that yielded any positive significant results in Experiment 1. All other analyses either yielded non‐results or only voxels that were significantly anti‐correlated with behaviour, that is, where damage in a voxel was associated with less severe symptoms

	Non‐overlapping		Overlapping	
	LSC−	LSC+	LSC−	LSC+
Ground truth (70% signal)				
SVC−	30/30	30/30	29/30	29/30
SVC+	23/30	24/30	11/30	15/30
Ground truth (50% signal)				
SVC−	30/30	28/30	29/30	28/30
SVC+	8/30	9/30	1/30	2/30

Abbreviations: LSC−, without control for lesion size; LSC+, with control for lesion size; SVC−, no second variable control; SVC, with control for the second variable.

For 70% signal and non‐overlapping ground truth regions, Dice index was affected by an interaction of lesion size control and control for the second variable (*F*(1, 103) = 9.24, *p* < .01). A significant simple main effect was found for lesion size control given the second variable is not controlled for (*p* < .001), with a higher Dice index when lesion size is controlled, but not if the second variable is controlled for (*p* = .96). Also, it has been found that there is no effect of the control of the second variable given lesion size is not controlled for (*p* = .14), but a significant effect given lesion size is controlled for (*p* < .01), with a lower Dice index if the second variable is controlled for. For DCoM, the interaction also became significant (*F*(1, 103) = 7.14, *p* < .01). Control for lesion sizes significantly reduced DCoM given the second variable is not controlled for (*p* < .01), but no effect was found given the second variable is controlled for (*p* = .37). Given lesion size is not controlled, control for the second variable also significantly reduced DCoM (*p* < .05), but not given lesion size is controlled for (*p* = .16).

For 70% signal and overlapping ground truth regions with a focus on the complete target region, Dice index was affected by an interaction of both variables (*F*(1, 80) = 11.19, *p* < .01). The simple main effect analysis revealed that control for the second variable decreased the Dice index both given lesion size is controlled for (*p* < .001) or given it is not controlled for (*p* < .05). Lesion size control increased the Dice index given the second variable was not controlled for (*p* < .001), but not given it was controlled for (*p* = .60). For DCoM, the interaction did not become significant (*F*(1, 80) = 2.87, *p* = .09), neither did a main effect for control for lesion size (*F*(1, 80) = 0.44, *p* = .51). However, a main effect for control of the second variable was significant (*F*(1, 80) = 7.04, *p* < .05), with higher DCoM with the control factor.

For 70% signal and overlapping ground truth regions with a focus on only the areas exclusive to the target ground truth, Dice index was again affected by an interaction of both variables (*F*(1, 80) = 4.75, *p* < .05). The simple main effect analysis revealed that control for the second variable decreased the Dice index given lesion size is controlled for (*p* < .01), but not given it is not controlled for (*p* = .79). Lesion size control increased the Dice index given the second variable was not controlled for (*p* < .001), but not given it was controlled for (*p* = .70). For DCoM, the interaction did not become significant (*F*(1, 80) = 2.26, *p* = .61), neither did a main effect for control for lesion size (*F*(1, 80) = 0.32, *p* = .57), nor a main effect for control of the second variable (*F*(1, 80) = 1.04, *p* = .31).

For 50% signal and non‐overlapping ground truth regions, Dice index was neither affected by an interaction between control for the second variable and lesion size (*F*(1, 71) = 0.01, *p* = .92), nor a main effect for control of lesion size (*F*(1, 71) = 0.42, *p* = .52). However, Dice Index was affected by control for the second variable (*F*(1, 71) = 11.44, *p* < .01), with a higher Dice index when the control was not applied. For DCoM, neither the interaction (*F*(1, 71) = 2.49, *p* = .12), nor main effects for the control of the second variable (*F*(1, 71) = 0.07, *p* = .79), nor control for lesion size (*F*(1, 71) = 0.14, *p* = .71) became significant.

### 3.3 Discussion

The control of the target behaviour by a second, correlating behavioural variable did not generally improve the validity of VLBM. More often, it even led to significantly less precise results than uncontrolled analyses. The drawbacks of such control could not be overcome by changing the focus of the analysis, that is, aiming to identify only brain regions that are exclusive to the target behaviour. On the contrary, most analyses improved with control for lesion size, and the condition with only control for lesion size was numerically the best in all conditions for both dependent variables.

Another finding was that analyses that were controlled for a second behavioural variable often resulted in non‐findings—or instead even negative, anti‐correlated significant findings. This almost never happened without such control (including mere control for lesion size). Control of the target behaviour by a second behavioural variable thus seems to be excessively conservative, and in some cases obscure anti‐correlated findings might appear as a statistical artefact.

## 4 EXPERIMENT 2: MAPPING OF COMMON BEHAVIOURAL FACTORS

### 4.1 Methods

The second experiment investigated the mapping of common behavioural factors as found by dimension reduction procedures. We hypothesised that common behavioural factors in neuropsychological behavioural data are not specific to actually shared cognitive functions, and thus the mapping of such factors can lead to artefacts. We therefore investigated the mapping of common factors in both actual behavioural symptoms and well‐controlled simulations. First, two behavioural variables that presumably do not share common cognitive functions and anatomical areas—primary motor function and visual spatial attention—were investigated. Second, symptoms based on non‐overlapping ground truth regions were simulated as in Experiment 1. In these simplified examples that were based on just two measures, we aimed to identify a possible common factor of both symptoms and to map this factor using VLBM.

For the analysis on real‐world data, we retrospectively identified and included 46 right hemisphere stroke patients that were tested and graded for both severity of hemiparesis of the upper limb and severity of spatial neglect. Primary motor skills were assessed using the Medical Research Council Grading, scoring the severity of upper limb hemiparesis on a scale between 0 and 5, with 0 being severe hemiplegia and 5 no hemiparesis at all. Visual spatial attention was assessed by the bells cancellation task (Gauthier et al., 1989) and the letter cancellation task (Weintraub & Mesulam, 1985). Both cancellation tests were analysed by assessing the Centre of Cancellation (Rorden & Karnath, 2010), a continuous score of the egocentric deficit in spatial neglect. Centre of Cancellation scores were then averaged for both tests and their polarity was inverted to mirror the poling of the hemiparesis score, with lower test scores indicating lower performance and more severe symptoms.

A principal component analysis using varimax rotation with Kaiser normalisation in SPSS was run to identify the first common factor of hemiparesis and spatial attention. Then, the scores for this first factor for each individual patient were obtained and mapped by VLBM. Mass‐univariate *t* tests both with permutation thresholding with 4,000 permutations at *p* < .05, and, as an additional post hoc analysis, with false discovery rate of *q* = 0.05 were performed, which also mirrors better VLBM parameters in several previous studies (e.g., Timpert et al., 2015; Verdon et al., 2010). Only voxels affected by at least four subjects were included into the analysis.

For the second analysis on simulated data, we chose 10 pairs of regions in the brain atlas used in Experiment 1 (Glasser et al., 2016; Pustina et al., 2018) that were not overlapping and not adjacent. Thus, no shared neural correlates of simulated scores existed. The simulation strategy procedures were the same as in Experiment 1, and were based on the same 100 lesions. In short, for both areas, a symptom score was computed based on a linear relation between the amount of lesioned voxels in the area and the symptom, and some noise was added to obtain more realistic data. Symptoms were simulated with 70% signal and 30% noise in order to ensure a high correlation between all pairs of simulated data. Doing so, we obtained correlations in the same range as found between the data on hemiparesis and spatial attention. Simulated scores correlated on average by *r* = .45 (SD = 0.12). Principal components were identified as described for the real‐world data. The VLBM analyses were performed with permutation thresholding with 1,000 permutations at *p* < .05 either with or without correction for lesion size. Only voxels affected by at least 10 lesions were analysed.

### 4.2 Results

**Figure 6:**
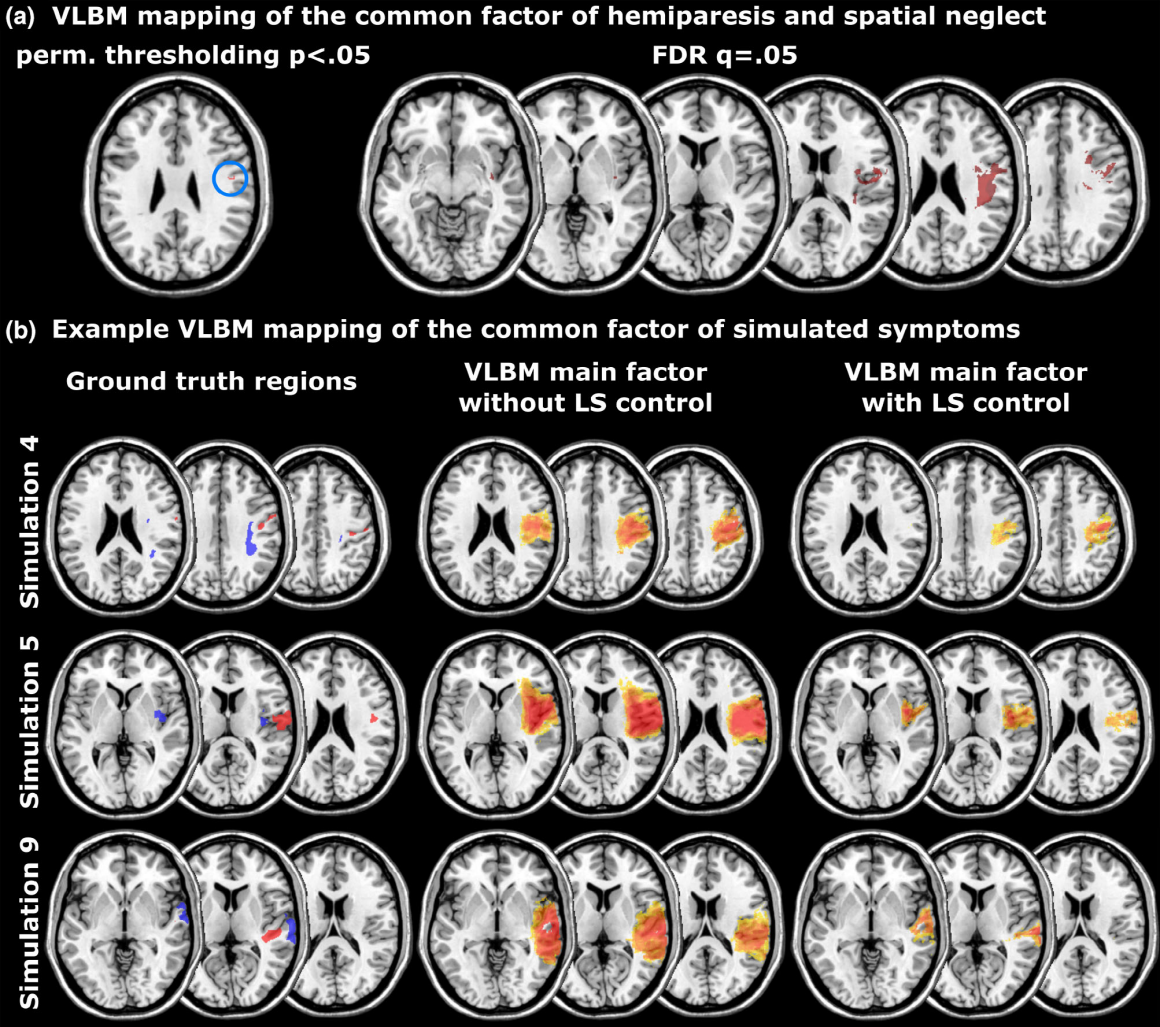
Results of Experiment 2, showing (a) the mapping of a common factor of hemiparesis and spatial neglect, and (b) example results of the mapping of common factors in simulated behavioural data that were based on non‐overlapping, non‐adjacent ground truth regions. Analyses in (b) were either controlled for lesion size (LS) or not controlled for lesion size

A medium Pearson correlation between hemiparesis and spatial neglect of *r* = .51 (*p* < .001) was found. The first factor in the principal component analysis explained 75.3% of variance and had an eigenvalue of 1.51. The VLBM corrected by permutation thresholding only identified 23 significant voxels, however the FDR‐corrected analysis found 10,476 significant voxels to be the neural correlate of this factor (Figure 6(a)). Both results, however, disappeared when a control for lesion size was applied.

For simulated data, the first factor explained on average 72.3% (SD = 6.1) of variance. All VLBM analyses, both controlled and uncontrolled for lesion size, found significant results. Analyses not controlled for lesion size yielded on average 72,170 (SD = 17,500) significant voxels, and analyses controlled for lesion size yielded on average 19,348 (SD = 11,661) significant voxels. Example results are shown in Figure 6(b).

### 4.3 Discussion

We chose the two variables primary motor function and visual spatial attention, for which we a priori assumed that no common neural correlates should exist. Nevertheless, VLBM of the common factor of hemiparesis and visual spatial neglect revealed several areas to be its common ‘neural correlate’. Thus, the finding appears to be a false positive finding. Note that a possible objection against the real‐world example could be that primary motor skills actually share common cognitive processes with visual spatial attention that are damaged in so‐called ‘motor neglect’. Motor neglect is indeed a post‐stroke symptom of attention in motor tasks, however it is by no means a link between hemiparesis and visual spatial neglect, but rather a highly specific symptom itself (e.g., Kojović & Bhatia, 2019).

The finding in real‐world data was further supported by simulations. When symptoms were simulated based on the damage status of non‐overlapping, non‐adjacent ground truth regions, VLBM still found neural correlates to underlie a common factor in all analyses. The mapping of common factors underlying multiple behavioural variables thus appears to be prone to false positive findings.

## 5 GENERAL DISCUSSION

We investigated two common strategies that are used to investigate the multivariate character of neuropsychological post‐stroke behaviour. In both cases, we observed that such approaches do not live up to the complexities of lesion‐behaviour relationships, and potentially impair the precision of topographical results, creating false‐positive artefacts.

**Figure 7:**
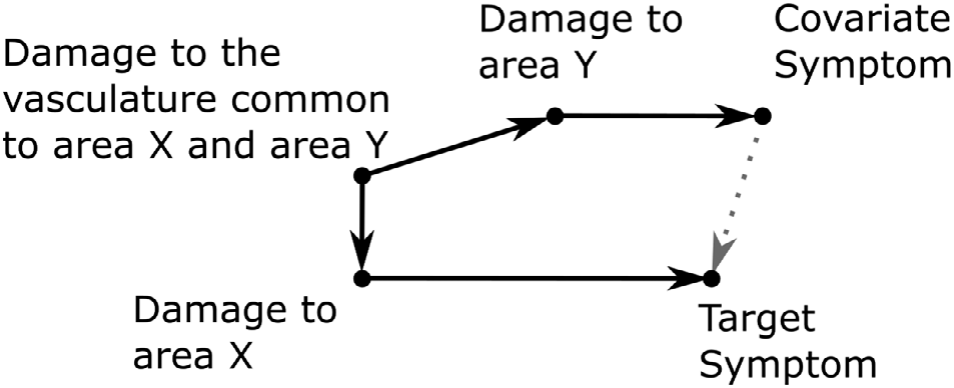
A graphical causal model of lesion‐deficit relations in a situation where damage to a sub‐region of the vasculature can induce damage to two distinct areas. Damage in each of the two areas can lead to a post‐stroke symptom. One of those is the target symptom that is supposed to be investigated by voxel‐based lesion‐behaviour mapping (VLBM), central to the study is the causal relation between damage to area X and the target symptom, that is, we are interested in mapping the neural correlates of the target symptom. The other symptom highly correlates with the target symptom, and one might consider including it as a covariate into the analysis. However, without any direct causal effect of the covariate symptom on the target symptom (dotted grey line), no actual confounding would be present at all, and covariate control would bias the analysis

Nuisance control of a second variable in VLBM did not improve the precision of topographical results. Should behavioural data in lesion mapping therefore never be controlled for other behavioural variables? The present study suggests that controlling for variables that do not directly affect the mapped target variable is unfavourable and should be avoided. However, and this is the reason why we cannot provide a definitive answer, there are situations where a secondary behaviour directly affects the measurement of the target variable. As mentioned in the Introduction, deficient cognitive processes that are not intended to be mapped might directly influence the target variable. A prime example is any language‐based assessment of cognitive abilities that are not thought to be related to language. Aphasia might hinder task performance, and thus not only the cognitive deficit of interest, but also some language skills which are assessed with the task. If we look at neuropsychological deficits such as apraxia (being affected by aphasia) or extinction (being affected by spatial neglect), the control for a secondary variable might offer more merits than disadvantages. In such cases, and with a clearly formulated theoretical background, secondary variable control might be favourable. On the other hand, our present results demonstrate that any variable control which is performed without such theoretical basis should be avoided. A better understanding of why covariate control in lesion‐behaviour mapping can be disadvantageous can be obtained by causally modelling the relations between variables (Pearl, 1996; Pearl & Mackenzie, 2018). If, and only if, causal relations between variables fulfil the so‐called ‘back‐door‐criterion’, covariate control is appropriate (Pearl et al., 2016; Pearl & Mackenzie, 2018). This is fulfilled if there is an indirect causal path from the dependent variable leading into the independent variable (Pearl & Mackenzie, 2018, p. 158). If we look at an individual statistical test that investigates the relation between damage to an area X and a target symptom, and if we assume that a covariate symptom is co‐occurring with the target symptom due to shared vasculature, then causal relations could be graphically modelled as in Figure 7. In that situation, there is no back‐door path from the target symptom to the damage in area X, and thus by definition, there is no confounding present. If we control for the covariate symptom in this situation, we would end up controlling for the variance that we actually want to measure. However, when we add a causal link from the covariate symptom to the target symptom (the dotted grey line in Figure 7)—for example, if a covariate symptom such as aphasia unintentionally affects our target symptom—then a case of confounding is present, and covariate control would close the confounding back‐door path. Note that causal models are likely not suited to explain a full mass‐univariate analysis and its complexity. However, given the assumptions visualised in Figure 7, it can be shown that some ways of covariate control in lesion‐behaviour mapping can be disadvantageous.

As outlined above, a rationale behind controlling for secondary behavioural variables might be to obtain a ‘pure’ behavioural variable, while non‐controlled variables are expected to be not only related to the neural correlates of interest, but also to other brain regions due to systematic collateral damage. This methodological strategy, that has been used intuitively by many researchers, is mirrored by more recent discussions about the validity of lesion‐behaviour mapping. It has been shown that collateral lesion damage between voxels induces spatial biases in lesion‐behaviour mapping methods (Inoue et al., 2014; Mah et al., 2014; Sperber et al., 2019). As we have observed in the present experiment, the control for secondary variables is not a solution to this problem. Instead, an unspecific control for lesion size seems to be a better option for control here. Such control, although imperfect (see Xu et al., 2018, for a critical discussion), appears to partially counteract spatial biases (see also Sperber & Karnath, 2017), but will of course only have an effect if there is a relation between lesion size and symptom at all. This was the case for the simulations in the present study, but it is not necessarily the case in all real deficits (see DeMarco & Turkeltaub, 2018).

Experiment 2 has shown that the shared variance of neuropsychological variables does not necessarily represent shared cognitive functions. Furthermore, VLBM might associate such shared variance with brain areas, although shared neural correlates of the original variables do not exist. These findings, however, do not discredit such an approach per se. The central question here is if the common factor of the variables really represents shared cognitive functions. If this is truly the case, we see no objection against mapping it, and VLBM would be a reasonable and powerful tool to identify such shared neural correlates. A close look at dissociation patterns might be helpful to get a deeper understanding of how different cognitive functions anatomically relate to each other. A more fail‐safe alternative, however, might be—if possible—to find a behavioural task that directly assesses the shared cognitive function.

Thus, while we have shown that the above methods can be disadvantageous when used in VLBM, this does not mean that they must do so in any analysis. In fact, we believe that the presence of a strong theoretical background is a crucial point not only in previous studies using these methods but also in future experiments planning to use such strategies. The VLBM method itself performs a hypothesis‐free parametric mapping. Without a strong theoretical background utilising the methods that were investigated in the present article, this can be fatal. For example, researchers might misjudge that two behavioural variables partially share neural correlates. They might find putative neural correlates of the common factor, although they do not truly exist. Or, after controlling for a secondary variable, researchers might obtain counter‐intuitive findings of areas where damage negatively correlates with the behavioural deficit. While we have shown that this can be a methodological artefact that regularly arises after controlling for a second variable, the researchers might interpret this finding post hoc as brain areas that inhibit beneficial plastic reorganisation of functional brain anatomy. On the other hand, if a strong theoretical basis is present, the statistical methods investigated in the present paper can indeed provide a powerful tool to investigate human brain architecture and how different cognitive modules relate to each other.

The present study focused on mass‐univariate VLBM. In recent years, multivariate lesion‐behaviour mapping methods (MLBM) which model structural lesion data (but not behavioural data) in a multivariate way were established (Pustina et al., 2018; Zhang et al., 2014). These methods also seem to suffer from biases induced by systematic collateral damage (Sperber et al., 2019), which are likely a main reason for the findings of the present paper. Thus, MLBM will likely suffer the same limitations as VLBM when behavioural variables are controlled for other variables or if shared variance of variables is mapped.

A limitation of the present study is the limited ecological validity of simulations (Sperber & Karnath, 2018). While simulation‐based validation of lesion‐behaviour mapping methods is well‐controlled and allows high flexibility in investigating different methodological factors, it is not clear to what extent findings can be transferred to real data. Lesion‐deficit relations in simulations are over‐simplified, and choosing a certain strength of lesion‐behaviour relation might not be representative for all actual post‐stroke deficits. However, the present study did not aim to find an optimal method, but instead investigated limitations of methods. Very likely, limitations of VLBM in a simulation setting will also be in some way present in real VLBM analyses, and the present study has shown that these limitations are inherent to the method, as they even exist in the over‐simplified simulation setting.

## 6 CONCLUSION

Shared variance between post‐stroke behavioural variables is ambiguous and can have different reasons. Thus, statistical procedures that in any way consider this variance are prone to artefacts. Overall, the multivariate character of behavioural data in lesion‐behaviour mapping studies poses special challenges, and lesion‐behaviour mapping studies that aim to capture this character require a solid theoretical ground to provide valid results. If this is the case, lesion‐behaviour mapping using such statistical methods is a powerful tool to investigate brain architecture. However, when in doubt, it might be better to not use them, and instead researchers might rather focus on a simple, but well‐elaborated study design to control for possible confounds of the behavioural target variable or to identify brain areas underlying common cognitive functions.

## CONFLICT OF INTEREST

There are no conflict of interest to report.

## Data Availability

The datasets generated and analysed during the current study are not publicly available due to the data protection agreement of the Centre of Neurology at Tübingen University (approved by the local ethics committee) signed by the participants. The agreement covers data storage for a duration of 10 years at the Centre of Neurology at Tübingen University. They are available via the corresponding author as well as the local ethics committee (ethik.kommission@med.uni‐tuebingen.de) on reasonable request.
